# Prevalence and Intensity of Soil-Transmitted Helminths and Associated Factors among Adolescents and Adults in Bibugn Woreda, Northwest Ethiopia: A Community-Based Cross-Sectional Study

**DOI:** 10.1155/2021/7043881

**Published:** 2021-12-23

**Authors:** Abrham Goshu, Getaneh Alemu, Animen Ayehu

**Affiliations:** ^1^Bibugn Primary Hospital, Amhara National Regional Health Bureau, Bahir Dar, Ethiopia; ^2^Department of Medical Laboratory Science, School of Health Sciences, College of Medicine and Health Sciences, Bahir Dar University, Bahir Dar, Ethiopia

## Abstract

**Background:**

Soil-transmitted helminths are a common public health problem in Ethiopia, affecting all age groups. However, epidemiological studies and interventions primarily target school-age children, despite the fact that 44.6 million adults live in endemic areas. Hence, data on the prevalence and intensity of soil-transmitted helminths infections and associated factors among adolescents and adults helps to expand interventions.

**Objective:**

The aim of the study was to assess the prevalence and intensity of soil-transmitted helminths and associated factors among adolescents and adults in Bibugn Woreda, East Gojjam, Northwest Ethiopia.

**Methods:**

A community-based cross-sectional study was conducted in Bibugn Woreda from February to September 2021. Using multistage proportionate sampling technique, 641 adolescents and adults were enrolled in this study. Data on sociodemographic characteristics and factors associated with helminthic infections was collected using structured questionnaire prepared in Amharic and administered through face-to-face interview. Parasite detection in stool samples was performed using modified formol-ether concentration and Kato-Katz techniques following standard protocols. Data was entered and analyzed using Statistical Package for the Social Sciences software version 25. Multivariable logistic regression was used to assess factors associated with soil-transmitted helminths infections. *P* value <0.05 was considered as statistically significant.

**Results:**

The overall prevalence of soil-transmitted helminths infection was 20.9% (134/641). The most detected parasite was *Ascaris lumbricoides* (12.5%), followed by hookworm species (7.5%) and *Trichuris trichiura* (1.1%). Fecal egg counts revealed that 96.5% (112/116) of the infections were with light parasite intensity, while 3.5% (4/116) were with moderate parasite intensity. Family size >5 (AOR = 1.866; 95% CI: 1.221–2.853; *P*=0.004), absence of latrine (AOR = 3.675; 95% CI: 1.599–8.449; *P*=0.002), and no habit of hand washing before meal (AOR = 2.622; 95% CI: 1.073–6.405; *P*=0.034) were significantly associated with soil-transmitted helminths infections.

**Conclusion:**

There was moderate prevalence of soil-transmitted helminths among adolescents and adults with predominance of *A. lumbricoides.* Family size greater than five, absence of latrine, and no hand washing habit before meal predisposed adolescents and adults for soil-transmitted helminths. The existing school-based interventions should expand to address adolescents and adults.

## 1. Background

Soil-transmitted helminths (STHs) are nematode worms infecting humans through either parasite eggs or larvae from the soil [1]. *Ascaris lumbricoides (A. lumbricoides)*, *Trichuris trichiura (T. trichiura)*, and hookworm species (*Necator americanus* and *Ancylostoma duodenale*) are grouped under STHs [2, 3]. They are highly prevalent in developing countries, where there is poor socioeconomic status, inadequate safe water supply, poor environmental sanitation, and poor personal hygiene [4–7]. *Ascaris lumbricoides* and *T. trichiura* commonly occur in both rural and urban environments. In contrast, hookworm species infections are predominantly found in rural communities [8]. *Ascaris lumbricoides* and *T. trichiura* infections are acquired via ingestion of embryonated eggs from contaminated soil, while hookworm species infect humans when the filariform larva actively penetrates through the intact skin [9].

The main clinical outcomes of soil-transmitted helminths among adolescents and adults are intestinal bleeding which leads to anemia because of chronic blood loss due to the infestation of the intestine by adult hookworms [10] and small bowel or bile duct obstruction due to the accumulation of adult worm in the bowel. This can cause severe abdominal cramping and vomiting (*A. lumbricoides*) [11] and anemia and chronic dysentery due to mucosal and rectal bleeding caused by *T. trichiura* [12].

Soil-transmitted helminths are among the most common and persistent parasitic infections worldwide [13, 14]. Their distribution is more common in South America, China, Southeast Asia, India, and sub-Saharan Africa [2]. They cause an estimated 5.3 billion cases worldwide, 39 million disability-adjusted life-years lost (DALYs), and more than 100 thousand deaths per year. Around 4.2, 3.19, and 3.2 billion adults are at risk of *A. lumbricoides*, hookworm species, and *T. Trichiura* infection, respectively, of which 812, 584, and 514 million are infected by respective parasites [15].

In Ethiopia, there are 26, 21, and 11 million cases of *A. lumbricoides*, *T. trichiura*, and hookworm species infections, respectively [16]. The national prevalence of STHs constitutes serious public health problem with estimated nationwide prevalence of 28.8% [17].

Different factors contribute to STH infection among adults and adolescents. These include occupation, in which adults are more engaged in agricultural activity, which remains a common denominator for hookworm species infection. Using night soil as a fertilizer is also another factor contributing to STH infection; as adults are the working manpower and perform the day-to-day activity of the household, they are easily infected with STHs. Other predisposing factors for STHs infections are poverty, sanitation, large family size, poor handwashing practice, and walking barefoot [18].

In Ethiopia, 53.6 million people require STH treatments, of which 31.3 million are adults [19, 20]. Soil-transmitted helminths pose direct medical and economic impact in adolescents and adults. Moreover, these segments of the population serve as reservoirs for continued transmission to children, the most vulnerable groups. Despite this, epidemiological studies and interventions primarily target school-age children in Ethiopia. Similarly, factors contributing to transmission of STHs in the community are not well addressed in Ethiopia generally and in Bibugn Woreda particularly. Even either the available studies on adolescents and adults are conducted among people with concurrent diseases like tuberculosis (TB) and human immune deficiency virus (HIV) or data are at health institution level. Such data do not exactly reflect STHs distribution in the community. Community level epidemiological data among adolescents and adults will be an important input for targeted intervention. Therefore, in this cross-sectional study, we aimed to assess the soil-transmitted helminths infection and its associated factors among adolescents and adults in Bibugn Woreda, Northwest Ethiopia.

## 2. Methods

### 2.1. Study Design, Period, and Area

A community-based cross-sectional study was conducted in Bibugn Woreda from February to September 2021. Bibugn is one of the woredas in East Gojjam zone, which is bordered on the south by Sinan, on the west by Dega Damot, on the northwest by Goncha, and on the east by Hulet Ej Enese. The woreda is 381 km far from Addis Ababa, the capital city of Ethiopia, 145 km from Bahir Dar, and 81 km from Debre Markose. It has a total of 18 kebeles. The weather condition of the woreda includes kola, Woyna Dega, Dega, and wurch. The woreda's annual temperature and rainfall are 9–24°C and 1200–1800 mm, respectively. The altitude ranges from 1500 to 4160 m above sea level. The majority of the population economy is based on agriculture. Since the study area is rural, use of night soil as a fertilizer and work in bare foot are common practices.

### 2.2. Sample Size Determination and Sampling Technique

#### 2.2.1. Sample Size Determination

The sample size was determined using single population proportion formula by taking the proportion of infected adults as 50% because of the absence of data on the STH prevalence at the community level, considering 95% confidence level (*zα*/2 = 1.96) and 5% marginal error (*d* = 0.05).

Therefore, the sample size was calculated using the following formula:

(1) n=Zα2^2×p1−pd2,

where *n* is the minimum sample size, Z*α*/2 is the confidence interval level 95% (1.96), *p* is the prevalence of STHs taken as 50%, and *d* is the margin of error of 5%.

(2) $$\begin{array}{c} \left(1.96\right)2\times \left(0.5\right)\times \frac{\left(1-0.5\right)}{{\left(0.05\right)}^{2}}=384,\\ n=384+\text{nonresponse rate}\left(10\%\right)=384+38.4=422. \end{array}$$

Since study participants were selected by multistage sampling technique, a design effect of 1.5 was used to get a final sample size of 663.

#### 2.2.2. Sampling Technique

**Figure 1:**
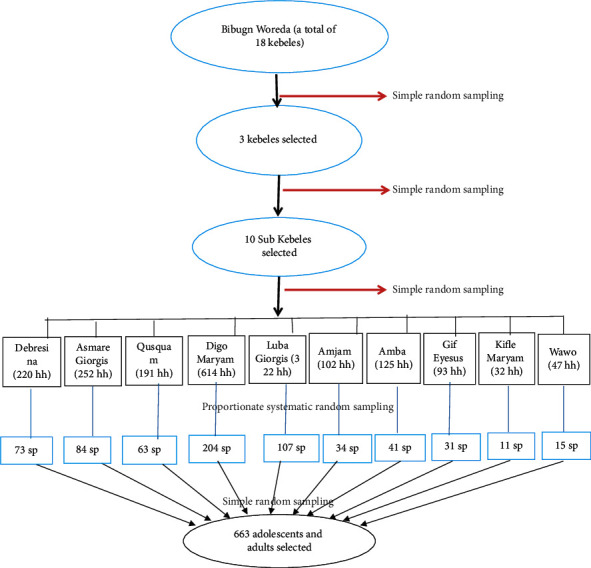
The flow of study participants' selection.

From eighteen kebeles in Bibugn Woreda, three kebeles were selected by simple random sampling method. Then, from the three kebeles, ten subkebeles were selected by simple random sampling method. From 1,998 households in the ten subkebeles, the number of participants in each subkebele was allocated proportionally. Then, the first household was selected by simple random sampling method and the next household was selected by systematic random sampling technique. For example, from Debresina subkebele (220 households), 73 households were selected by proportional allocation, and 73 study participants were from Debresina subkebele. Likewise, the remaining study participants from each subkebele were selected. Finally, 663 study participants were included in the study, by selecting one study participant from each household (Figure 1).

#### 2.2.3. Inclusion Criteria

Adolescents and adults who gave consent, stayed in the Woreda for at least 6 months, and volunteered to participate were included in the study.

#### 2.2.4. Exclusion Criteria

Adolescents and adults who took anthelminthic drugs 2 months prior to or during data collection time and ill adolescents and adults who were unable to respond to research questions were excluded from the study.

### 2.3. Data Collection and Processing

#### 2.3.1. Questionnaire Data

The study participants were oriented about the aim of the study and how to collect and provide sufficient stool specimen by trained laboratory technician and the principal investigator. The Amharic version of the questionnaire was administered by trained laboratory technician through face-to-face interview. Data on sociodemographic characteristics and water, hygiene, and sanitary conditions were collected using a structured questionnaire.

#### 2.3.2. Stool Collection and Processing

A 25-milliliter clean, dry, and tightly screwed stool cup was given for each study participant and they were advised to collect about 2 grams of morning stool. The stool sample was collected by trained laboratory technician and transported in cool chain to nearby health facilities. The sample was processed 1st for Kato-Katz (KK) and then modified formol-ether concentration (FEC) by trained laboratory technologist and the principal investigator to detect the prevalence and intensity of STHs.

In Ritchie's (modified formol-ether concentration) method, first 2.5 ml formalin was added to Ritchie's tube followed by a 1 ml diethyl ether. About 0.5 grams of fresh stool sample was added to Ritchie's tube. Then, the mixture was mixed and centrifuged at 1000 revolutions per minute for 3 minutes. After centrifugation, the top three layers were discarded and the sediment was mixed. Finally, one drop of the sediment was added on a microscope slide and the ova of parasites were examined using a microscope by trained laboratory technologist and the principal investigator.

In Kato-Katz thick smear technique, a fresh stool sample was pressed through a sieve (mesh) to remove large particles. 41.7 mg of sieved stool sample was transferred to the template put on a microscope slide. Then, the template was removed from the microscope slide and the stool was covered with cellophane immersed with glycerol-malachite green solution for 24 hrs. The cellophane was pressed with another microscope slide to form a smear. Then, the pressing slide was removed sideway to prevent the detaching of the cellophane from the smear. From each study participant, two KK slides were prepared. Slides were examined under the microscope within 45 to 60 minutes of smear preparation to prevent losing of hookworm parasites. The total number of eggs was counted and multiplied by a factor of 24 to obtain eggs per gram (epg) of feces.

### 2.4. Quality Control

Training was given for laboratory technicians about questionnaire data and stool sample collection, as well as examination procedures. The completeness of the questionnaire and quantity of the sample were checked daily by the principal investigator. Data collectors were supervised during data collection. For egg quantification, each prepared smear was examined by a laboratory technologist and a principal investigator, and the average result was taken.

### 2.5. Data Analysis

Data was entered and analyzed using Statistical Package for the Social Sciences software version 25 (SPSS). Descriptive statistics were used to describe study participants and to compute prevalence of parasites, while binary logistic regression was used to assess factors associated with STHs infections. A variable with *P value* less than 0.2 was entered into multivariable logistic regression. Then, an association between variables and STHs infection was considered significant if *P* value < 0.05 at 95% confidence level.

## 3. Results

### 3.1. Sociodemographic Characteristics of Study Participants

Among a sample size of 663, complete data was collected from 641 participants, giving a response rate of 96.7%. No eligible person was available at home in 15 households during data collectors' visit, while 7 participants were unable to provide stool sample. In case of unavailability of eligible persons, data collectors have revisited the house once again. Study participants were aged between 15 and 72 years, with a mean age of 37.5 ± 0.537 years. Most study participants were from Digo Kanta kebele, 43.9% (280/641). From the total study participants, 63.3% (406/641) were males and the remaining 36.7% (235/641) were females. 292 (45.6%) of the study participants were aged between 31 and 50 years and 65.7% (421/641) were illiterate. Most of the study participants (84.4%) were farmers (Table 1).

### 3.2. Prevalence of Soil-Transmitted Helminths

In this study, the overall STHs prevalence was 20.9% (134/641) with 95% CI of 17.9–24.3. The prevalence of *A. lumbricoides,* hookworm species, and *T. trichiura* was 12.5% (95% CI: 10.7–15.1), 7.5% (95% CI: 5.2–9.4), and 1.1% (95% CI: 0.3–1.9), respectively. Among STH-infected participants, 0.8% (1/134) were with double infection (*A. lumbricoides* and hookworm species). Among kebeles included in the study, the highest STHs prevalence of 31.2% (39/125) was recorded in Debresina Asmare (Table 2).

### 3.3. Intensity of Soil-Transmitted Helminths

The majority of STH-positive study participants had light-intensity infections. From Kato-Katz-detected STHs parasites, 96.5% (112/116) and 3.5% (4/116) of infections were of light and moderate intensity, respectively. There were no heavy-intensity infections recorded in the study area (Table 3).

### 3.4. Factors Associated with Prevalence of Soil-Transmitted Helminths

In the bivariate logistic regression, educational status, finger nail status, family size, toilet availability, and habit of washing hands before meal were factors with *P* value < 0.2. Among those, family size >5 (AOR = 1.866; 95% CI: 1.221–2.853; *P* = 0.004), absence of latrine (AOR = 3.675; 95% CI: 1.599–8.449; *P* = 0.002), and no habit of hand washing before meal (AOR = 2.622; 95% CI: 1.073–6.405; *P* = 0.034) were significantly associated with STHs infection (Table 4).

## 4. Discussion

In the present study, the overall prevalence of STHs was 20.9% (95% CI: 17.9–24.3). This is in line with STH prevalence reported from coastal area of Kenya (22.5%) [21]. On the contrary, it is lower than the prevalence of STHs reported from Bench Maji Zone, Ethiopia (69.5%) [22], central region of Cameroon (51.5%) [23], and the national STH prevalence in Ethiopia (28.8%) [17]. This difference might be due to variation in weather conditions where there are hot, humid, and wet weather conditions in central Cameroon (average annual temperature of 24.2°C) and Bench Maji Zone (average temperature of 20°C) which is not similar to that of the study area, where there is cold weather condition with 9°C to 24°C temperature and altitude of 1500 to 4160 m above sea level, especially in Ded Eyesus kebele, which is not suitable for the survival of hookworm species which in turn decrease the overall STHs prevalence. On the other hand, the present STHs prevalence is higher than study results from rural Kenya (15.7%) [24] and Zambia (9.8%) [25]. This might be due to variations in diagnostic method where only Kato-Katz method and only formol-ether concentration technique were used in Zambia and Kenya, respectively. In the present study, we used both Kato-Katz and modified formol-ether concentration techniques which might increase parasite detection.

In this study, the predominant STH parasite detected was *A. lumbricoides* (12.5% (95% CI: 10.7–15.1)), followed by hookworm species (7.5%) (95% CI: 5.2–9.4) and *T. trichiura* (1.1%) (95% CI: 0.3–1.9). The prevalence of *A. lumbricoides* is lower than the national prevalence of Ethiopia (37%) [26], Southwest Nigeria (55.2%) [27], and Jimma town, Ethiopia (50%) [28]. This might be due to study time variation in which there have been many intervention activities done in the country. This might decrease parasite prevalence through implementation of health extensions' house to house education, open defecation free environment, and mass drug administration program among school-age children in the country. On the contrary, the present study's prevalence is higher than the prevalence reported from Tanzania (8.3%) [7], Bench Maji Zone, Ethiopia (8%) [22], Harbu Town, Ethiopia (0.8%) [29], Zambia (7%) [25], and rural Kenya with prevalence of 6.1% [24]. This might be due to diagnostic method variation (only Kato-Katz methods in Tanzania, Harbu Town, and Zambia, direct wet mount and Kato-Katz in Bench Maji Zone, and only concentration technique in rural Kenya). In contrast, the present study's finding used both modified FEC and Kato-Katz methods, which increases parasite detection.

Prevalence of hookworm species (7.5%) in the present study is in line with the study findings from Malaysia with prevalence of 8.6% [30] and Bench Maji Zone, Ethiopia (6%) [22]. However, it is lower than the national prevalence of Ethiopia (16%) [26], Jimma town, Ethiopia (19.7%) [28], and Bahir Dar, Northwest Ethiopia (35.2%) [16]. This might be due to a cold weather condition (9°C to 24°C temperature) and high altitude (1500 to 4160 m above sea level) which reduce hookworm distribution and transmission in Ded Eyesus kebele in the present study. On the other hand, the prevalence is higher than study findings in Harbu Town (1.6%) [29] and in central Kenya (0.2%) [31]. This might be a result of laboratory method difference, where there is only Kato-Katz method in central Kenya and Harbu Town.

The prevalence of *T. trichiura* (1.1%) in this study is in line with study findings from rural southwestern Kenya (0.6%) [24] and Harbu Town, Northeastern Ethiopia (0.4%) [29]. On the other hand, the present study's finding is lower than study findings in Jimma town with 73.6% prevalence [28], 30% national prevalence of Ethiopia [26], and in Malaysia with the prevalence of 49.5% [30]. This might be a result of intervention activities done like deworming program in school-age children and creation of open defection free environment in the community.

According to the WHO classification criteria [32], there were 100% light-intensity infections of *A. lumbricoides* recorded in the study area. This is similar to the finding from Kogi State, Nigeria, where there was 100% light-intensity infection [33]. However, it is lower than study findings in Bench Maji Zone, Southwest Ethiopia, where 70.7% and 29.3% were light- and moderate-intensity infections, respectively [22], and Bushulo village, Southern Ethiopia, where there were 70.5%, 24.3%, and 5.1% of light-, moderate-, and heavy-intensity infections, respectively [34]. Similar to *A. lumbricoides, T. trichiura* had 100% light-intensity infection in the present study, which is consistent with study findings from Kogi State, Nigeria [33]. On the contrary, it is lower than findings from Bench Maji Zone, Southwest Ethiopia, where there were 62.2%, 36.1%, and 1.1% of light-, moderate-, and heavy-intensity infections of *T. trichiura* [22] and 93.7% and 6.3% of light- and moderate-intensity infections in Bushulo village, Southern Ethiopia [34]. This might be due to mass drug administration in school-age children which reduces the prevalence and intensity of *A. lumbricoides* and *T. trichiura*. On the other hand, there were 90% and 10% light- and moderate-intensity hookworm species infections in the present study's finding, which is similar to a study finding from Kogi State, Nigeria, where there were 98.2% and 1.8% light- and moderate-intensity hookworm species infections, respectively [33]. But the present study's finding is higher than study results in Bench Maji Zone, Southwest Ethiopia, where there was 100% light-intensity infection [22]. This might be due to single Kato-Katz diagnostic method used in Bench Maji Zone which could affect the prevalence and intensity of STH infections in contrast with double Kato-Katz diagnostic method in the present study's finding.

The present study revealed that having family size greater than five was significantly associated with increased prevalence of STHs infection. Study participants having family size greater than five were 1.87 times more likely to be infected by STHs than individuals having five family members or less. This finding agrees with study findings from Malaysia [30]. This is because if one of the family members is infected, there will be a probability of transmission to others. So as the family size increases, there is a probability to get at least one member infected, who in turn transmits the infection to others.

Adults and adolescents who had no toilet in their compound were 3.68 times more likely to acquire STHs than those who had toilet in their compound. This finding agrees with findings from Ethiopia [35]. This is because it minimizes parasite transmission from open defecation which is suitable for parasite distribution. The study also showed that no hand washing before meal was a significantly associated factor for the increased prevalence of STHs infection. Adults and adolescents who did not wash their hands before meal were 2.62 times more likely to be infected by STHs than adults and adolescents who washed their hands before meal. This is because poor hand washing habit before meal might increase chance of entry of parasites into individuals from contaminated drinking water and food.

Another important risk factor that contributed to the high prevalence of STHs infection among adolescents and adults in Bibugn Woreda was consumption of raw vegetables. The prevalence of STHs was higher in study participants who used raw vegetables than in participants who do not eat raw vegetables, but the difference was not statistically significant, which contradicts with previous study findings from Ethiopia [35]. In addition, STH prevalence was higher in males than in females, but the difference was not statistically significant, which contradicts with findings from coastal area of Kenya [21], Bench Maji Zone [22], and Bushulo village, Southern Ethiopia [34], where males were significantly at higher risk of STHs compared with females. This might be due to occupational difference as males are more engaged in field activities where the probability of being infected is relatively high. There were also other factors like age, educational status, occupational status, toilet utilization, toilet cover, habits of washing raw fruits and vegetables, and finger nail status which were not statistically significant with the prevalence of STHs in the present study. This might be due to response bias of study participants, which contradicts the ground truth, and there might be also undetermined confounders in the study area.

## 5. Conclusion

Moderate prevalence of STHs is recorded among adolescents and adults in the study area. Almost all STH-positive study participants in the study area had light-intensity infections. Large family size, absence of toilet, and no hand washing before meal were identified as predictors of STHs infection. Hence, deworming programs should be supplied in selected subkebeles of the study area once a year to stop the perpetuation of the transmission and reinfection of STHs, which leads to a reduction of the gain achieved by deworming children. Other measures like health education (through health extension workers and woreda health officers), modernization of sanitary facilities, and improved personal hygiene (through all community members) should also be considered.

## Figures and Tables

**Table 1:** Sociodemographic characteristics of study participants (*n* = 641) in Bibugn Woreda, East Gojjam, Ethiopia, from February to September 2021.

Variables	Category	Frequency	Percentage
Gender	Female	235	36.7
	Male	406	63.3

Age group in years	15–18	45	7.0
	19–30	187	29.2
	31–50	292	45.6
	>50	117	18.3

Address (kebele)	Debresina Asmare	125	19.5
	Digo Kanta	280	43.7
	Ded Eyesus	236	36.8

Educational status	Illiterate	421	65.7
	Primary school	111	17.3
	Secondary school	91	14.2
	College and above	18	2.8

Occupational status	Farmer	541	84.4
	Student	80	12.5
	Merchant	11	1.7
	Government employee	9	1.4

Family size	≤5	451	70.4
	>5	190	29.6

**Table 2:** Prevalence of STH infections in adolescents and adults (*n* = 641) by sociodemographic factors in Bibugn Woreda, East Gojjam, Ethiopia, from February to September 2021.

Variables	Category	Frequency	STH-positive N (%)
Gender	Female	235	45 (19.1)
	Male	406	89 (21.9)

Age group in years	15–18	45	10 (22.2)
	19–30	187	38 (20.3)
	31–50	292	61 (20.9)
	>50	117	25 (21.4)

Address (kebele)	Debresina Asmare	125	39 (31.2)
	Digo Kanta	280	65 (23.21)
	Ded Eyesus	236	31 (13.14)

Educational status	Illiterate	421	100 (23.8)
	Primary	111	18 (16.2)
	Secondary school	91	14 (15.4)
	College and above	18	2 (11.1)

Occupational status	Farmer	541	118 (22.0)
	Student	80	13 (16.3)
	Merchant	11	2 (18.2)
	Government employee	9	1 (11.1)

Family size	≤5	451	83 (18.4)
	>5	190	51 (26.8)

Total	641	134 (20.9%)	

**Table 3:** Intensity of STHs among adolescents and adults (*n* = 641) in Bibugn Woreda, East Gojjam, Ethiopia, from February to September 2021.

Parasite species	Intensity of infection		
	Mean (egg/gram)	Light number (%)	Moderate number (%)
*A. lumbricoides*	758.61	69 (100)	0
Hookworm species	587.12	37 (90)	4 (10)
*T. trichiura*	328	6 (100)	0

**Table 4:** The possible potential factors associated with STH infections among adolescents and adults (*n* = 641) in Bibugn Woreda, East Gojjam, Ethiopia, from February to September 2021.

Variables		Examined	Positive for STHs	Statistics			
			N (%)	COR (95% CI)	*P* value	AOR (95% CI)	*P* value
Gender	Female	235	45 (19.1)	1			
	Male	406	89 (21.9)	1.19 (0.79–1.77)	0.406		

Age in years	19–30	187	38 (20.3)	1			
	15–18	45	10 (22.2)	1.12 (0.51–2.46)	0.777		
	31–50	292	61 (20.9)	1.04 (0.66–1.63)	0.881		
	>50	117	25 (21.4)	1.07 (0.60–1.88)	0.827		

Educational status	Illiterate	421	100 (23.8)	2.49 (0.56–11.03)	0.229	1.77 (0.40–7.91)	0.455
	Primary	111	18 (16.2)	1.55 (0.33–7.33)	0.581	1.01 (0.21–4.89)	0.987
	Secondary	91	14 (15.4)	1.45 (0.30–7.04)	0.641	1.09 (0.22–5.37)	0.912
	College	18	2 (11.1)	1			

Occupational status	Farmer	541	118 (22.0)	2.23 (0.28–18.02)	0.451		
	Student	80	13 (16.3)	1.55 (0.18–13.49)	0.690		
	Merchant	11	2 (18.2)	1.78 (0.13–23.52)	0.662		
	Employees	9	1 (11.1)	1			

Family size	≤5	451	83 (18.4)	1		1	
	>5	190	51 (26.8)	1.63 (1.09–2.43)	0.017	1.87 (1.22–2.85)	0.004

Toilet availability	Yes	584	109 (19.0)	1		1	
	No	57	25 (43.9)	3.40 (1.94–5.98)	0.001	3.67 (1.60–8.45)	0.002

Toilet cover	Yes	314	62 (19.7)	1			
	No	270	72 (26.7)	1.20 (0.82–1.76)	0.352		

Toilet utilization	Yes	564	104 (18.4)	1			
	No	20	5 (25.0)	1.47 (0.52–4.15)	0.462		

Water source	Bono	352	67 (19.0)	1.17 (0.14–10.23)	0.884		
	Stream	283	66 (23.3)	1.52 (0.18–13.25)	0.704		
	Well	6	1 (16.7)	1			

Finger nail status	Trimmed	320	60 (18.8)	1		1	
	Untrimmed	321	74 (23.1)	1.30 (0.88–1.90)	0.181	1.14 (0.76–1.71)	0.528

Hand washing habit before meal	Yes	618	123 (20.0)	1		1	
	No	23	11 (47.8)	3.69 (1.59–8.56)	0.002	2.62 (1.07–6.41)	0.034

Habit of using raw vegetables and fruits	No	262	51 (19.5)	1			
	Yes	379	83 (21.9)	1.16 (0.79–1.72)	0.456		

Habit of washing vegetables and fruits	Yes	332	72 (21.7)	1			
	No	49	11 (22.4)	1.04 (0.50–2.13)	0.921		

## Data Availability

The datasets analyzed during the current study are not publicly available due to institutional regulation but are available from the corresponding author upon reasonable request.

## Abbreviations

AOR: Adjusted odds ratio
CI: Confidence interval
COR: Crude odds ratio
EPG: Eggs per gram
FEC: Formol-ether concentration
KK: Kato-Katz
SPSS: Statistical Package for the Social Sciences
SSA: Sub-Saharan Africa
STH: Soil-transmitted helminths
WHO: World Health Organization.
